# Proposed Changes in the Physicians’ Mutual Benefit Association

**Published:** 1888-03

**Authors:** Samuel A. Stone

**Affiliations:** Richmond, Texas


### PROPOSED CHANGES IN THE P. M. B. A.

LETTER FROM DR. SAMUEL A. STONE, OF RICHMOND, TEXAS.

RICHMOND, TEXAS, JANUARY, 19, 1888.

*F. E. Daniel, M. D.*

DEAR SIR.—Cannot we have a different arrangement of the P. M. B. A. T? I do not see why we could not make it more effectual than at present. According to the death rate as it is at present, the association has now existed about three and a half years, and this is our fifth assessment; less than two in a year. Now I would prefer this plan;—taking as a standard only one hundred members (and I feel satisfied we could easily have at least 500 or 1000 members,) Tax each member $4, per annum for dues that is only one dollar per quarter, and in addition tax each member $5 per quarter, which would make $20 per annum for death benefits, which would amount to $2000 a year, and at the death rate we have had we would be able to pay to each of our brother's widows or dependents $1000, which would be an amount worth something, and be of some service to his family. The amount of money for dues I would pay to the secretary and treasurer, which would be $400 per annum, when the members of the association should increase to as many as two hundred I would increase the death benefit to $2000, and should the membership increase to five hundred, as I am satisfied it would, I would make the death benefit only $2,500, that should be the limit, not to exceed it, by these means. I feel sure we could soon have in the association a reserve fund. I would not permit the salary of the secretary and treasurer to exceed $1,000, and he should be a bonded officer under the control and supervision of a board of trustees, who might be elected from the members of the association, and at the same time as the State Medical association met, let there be a meeting of the association and transact such business as might come before it, and at that time order all death claims to be paid. In part I think the Benefit association should be a part of the State Medical association.

Doctor, think of these suggestions and let us see what we can do at the next meeting of the State Medical association when it meets in Galveston.

Yours Truly,

SAMUEL A. STONE.

[Per contra, it is urged that most physicians below fifty years of age have as much insurance as they want, and that the organization is a charatible one, for the benefit of older members who have not, and cannot get insurance; and that to increase the tax on the younger members would be to drive them out: The above suggestions are submitted in advance of the meeting which it is proposed to hold in Galveston during the time of the Medical convention. It is earnestly desired that members will attend; we must amend the Constitution and By-laws, and make the order more efficient, or let it go by the board. It is falling off, and seventeen prominent physicians who paid assessment No. 4 for Dr. Kilpatrick, failed to pay No. 5 for Mrs. Starly, and are dropped. The Secretary desires to make a report at this meeting; and it is necessary to elect a board of trustees—as the trustees all dropped out the first year.]

